# Xenogeneic collagen matrix versus connective tissue graft for the treatment of multiple gingival recessions: A systematic review and meta‐analysis

**DOI:** 10.1002/cre2.210

**Published:** 2019-06-30

**Authors:** Mohammed A. AlSarhan, Reham Al Jasser, Mohammad Abdullah Tarish, Anas I. AlHuzaimi, Hamad Alzoman

**Affiliations:** ^1^ Department of Periodontics & Community Dentistry, College of Dentistry King Saud University Riyadh Saudi Arabia; ^2^ Department of Preventive Dental Science ELM University Riyadh Saudi Arabia; ^3^ Capital Health Region Ministry of Health Kuwait Kuwait; ^4^ College of Dentistry King Saud University Riyadh Saudi Arabia; ^5^ Medical Services Ministry of Interior Riyadh Saudi Arabia

**Keywords:** collagen matrix, connective tissue graft, gingival recession, meta‐analysis, systematic review

## Abstract

A systematic review and meta‐analysis was performed to understand the efficacy of xenogeneic collagen matrix (CMX) compared with connective tissue graft (CTG) for the treatment of multiple adjacent gingival recessions (MAGRs). A literature search was performed for published randomized controlled trials in adult patients (≥18 years old) with Multiple Adjacent Miller class I and II gingival recessions (MAGRs). The assessments included recession depth, Recession width, complete root coverage, mean root coverage, probing depth, clinical attachment level, and keratinized tissue width. Pooled data were analyzed using fixed‐ and random‐effects models, and Forest plots were constructed. Heterogeneity within studies was calculated to assess publication bias. Four randomized controlled trials were included based on the eligibility criteria. Although the recession depth, complete root coverage, and mean root coverage were significantly lower with CMX (*p* = .017 and *p* = .001, *p* = .001, respectively), there was no statistically significant difference in the Recession width between CMX and CTG (*p* = .203). CMX showed significantly lower Probing Depth than CTG (*p* = .023); however, no significant difference in clinical attachment level (*p* = .060) and keratinized tissue width (*p* = .052) was observed between the groups. Owing to the heterogeneity in the included studies, firm conclusions cannot be drawn regarding the noninferiority of CMX compared with CTG. Long‐term studies are therefore needed to conclusively establish the relative efficacy of CMX in MAGR.

## 1 INTRODUCTION

Gingival recession (GR) can be defined as the apical shift of the gingival margin with respect to the cemento‐enamel junction leading to attachment loss with variable percentage of root surface exposure. It is a substantial problem affecting middle and older age groups (Cortellini & Bissada, 2018). GR is typically caused due to various factors including but not limited to periodontal disease, inflammation, incorrect occlusal relationships, thin periodontal phenotype, tooth eruption pattern, and mechanical trauma (Armitage, 1999). GR can be either localized or multiple adjacent gingival recession (MAGR) with or without loss of attached gingival tissue with ensuing tooth sensitivity due to exposed dentin. The exposed root surface is frequently associated with esthetic complaints, root hypersensitivity, mechanical root wear, cervical root caries, and difficulties to achieve optimal plaque control (Tonetti et al., 2014); these issues prompt patients to seek corrective treatments. The absence of adequate mucogingival complex can lead to localized inflammation predisposing to GR development (Ravipudi, Appukuttan, Prakash, & Victor, 2017).

Various surgical and nonsurgical options are available for the treatment of GR. When GR is minimal, adequate thickness of tissue, favorable plaque control, not affecting aesthetics or causing dentin hypersensitivity and/or root caries, no treatment is needed. However, deeper defects are managed by surgical techniques that have been proposed as treatment modalities for GR with various outcomes in accomplishing root coverage (Miller, 1985; Miller, 1988; Tatakis et al., 2015). Root coverage techniques for all types of GRs are performed either with the objective of increasing keratinized tissue (KT) alone or a combination of KT, tissue regeneration, and coronally advanced flap (CAF). Traditionally, in the presence of less KT near the recession sites, a soft tissue grafting such as connective tissue graft (CTG) along with CAF or free gingival graft are recommended. However, if the width of the attached gingiva is adequate, CAF can be used alone (Pini‐Prato et al., 2010). Also, gingival thickness has an impact on the presence of GR and the outcomes of root coverage procedures. Gingival thickness less than 1 mm had reduced root coverage compared with thick gingival flaps (Hwang & Wang, 2006). Whereas periodontal plastic procedures are often performed primarily to restore form and function of teeth and its associated gingival complex, the CAF together with CTG was found to provide and maintain complete root coverage in both short‐ and long‐term periods (Lops et al., 2015). Indeed, a meta‐analysis has indicated that CAF + CTG was more effective in root coverage at single GR with Miller class I and II compared to CAF alone or CAF + Guided Tissue Regeneration (Cairo, Nieri, & Pagliaro, 2014). However, this technique has some inherent challenges for patients with multiple recession defects especially when variation in root prominence, vestibular depth, and degree of recession is present. In addition, there is a degree of morbidity associated with CTG harvesting especially when the quantity of donor tissue is limited (Tavelli et al., 2018; Wessel & Tatakis, 2008). Tunneling (TUN) technique gained popularity with clinicians by offering minimally invasive surgery with acceptable root coverage and better esthetic outcomes. A recent review on the efficacy of TUN versus CAF indicated that the former was useful for both localized and MGARs, although, the latter was found to be associated with better root coverage (Tavelli et al., 2018).

Currently, several biomaterials are available to overcome the shortcomings of autogenous soft tissue grafts including enamel matrix derivative, acellular dermal matrix, barrier membranes, and collagen matrix (Abolfazli, Saleh‐Saber, Eskandari, & Lafzi, 2009; Scarano, Barros, Iezzi, Piattelli, & Novaes, 2009; Tatakis & Trombelli, 2000). Among these materials, the initial data evaluating xenogeneic collagen matrix (CMX) showed promising results in single recession defects. CMX is a bilayer composed of an outer compacted layer designed to hold the suture and protect the defect and the inner porous matrix that promotes quick stabilization of blood clot and encouraging rapid vascularization and tissue integration (Ghanaati et al., 2011; Rocchietta, Schupbach, Ghezzi, Maschera, & Simion, 2012). CMX has been shown to promote regeneration of keratinized gingiva in both the width and thickness not only around natural tooth but also around dental implants (Sanz, Lorenzo, Aranda, Martin, & Orsini, 2009). A recent review (Atieh, Alsabeeha, Tawse‐Smith, & Payne, 2016) reported that CMX had better outcomes than CAF alone in terms of root coverage. However, CMX performed less in overall clinical outcomes compared with CAF + CTG. CTG + CAF had a higher percentage of complete/mean root coverage and mean recession reduction than CMX. CMX showed higher mean root coverage, recession reduction, and gain in KT than CAF alone. No significant differences were reported in patient's aesthetic satisfaction between CMX and CTG. Nevertheless, these findings were mostly related to isolated Miller class I and II marginal recession because there were limited reports of using CMX at multiple adjacent sites.

The goal of this systematic review was to compare the efficacy of CMX with CTG for the treatment of multiple adjacent Miller's Class I and Class II gingival recession (MAGR) in terms of clinical parameters and patient‐related outcomes.

## 2 METHODS

### 2.1 Study design

This systematic review focusing on the effect of CMX versus CTG for the treatment of MAGR was reported based on the Preferred Reporting Items for Systematic Review and Meta‐Analysis guidelines (Moher, Liberati, Tetzlaff, & Altman, 2009).

### 2.2 Registration

The protocol was specified and registered with the International Prospective Register of Systematic Reviews (PROSPERO), registration number CRD42019119831.

### 2.3 Eligibility criteria for study inclusion

Randomized controlled trials published in English language and with a minimum follow‐up of 3 months were eligible to be included. Nonrandomized clinical trials, retrospective studies, cross‐sectional studies, case series, and case reports were excluded.

PICO framework was applied as below:

Population: Adult patients (≥18 years old) with MAGR.
Intervention: Collagen Matrix (CMX).
Controls: Connective Tissue Graft (CTG).

### 2.4 Outcomes

Primary variables: recession depth (RD), recession width (RW), complete root coverage (CRC), and mean root coverage (MRC).

Secondary variables: clinical attachment level (CAL), probing depth (PD), and change in keratinized tissue width (KTW).

Root coverage was defined as the change in GR at follow‐up with regard to RD, RW, CRC, and MRC; change in KT was identified when an alteration in the width of keratinized gingiva (mm) was found during follow‐up; changes in gingival margin‐pocket base were measured as PD (mm) during follow‐up; changes in CAL was defined as a gain or change in attachment level (mm) at follow‐up.

### 2.5 Information sources and search strategy

Comprehensive search strategies were established. MEDLINE (via PubMed), EMBASE, and CENTRAL databases were searched from the earliest records through December 2018. Unpublished studies, thesis, clinical trial registries, and reference lists were also searched. In addition, hand search for the past 5 years of relevant dental journals (International Journal of Periodontics and Restorative Dentistry, Journal of Clinical Periodontology, Journal of Periodontal Research, Journal of Periodontology, and Quintessence International) was carried out to identify potential papers. Details regarding the search terms are presented in Table 1.

**Table 1:** Summary of search terms used for literature extraction

(plastic surgery”[MeSH Terms]) OR “mucogingival surgery”[Text Word]) OR “surgery”[Text Word]) OR “graft*”[Text Word]) OR “regen*”[Text Word]) OR “coverage”[Text Word]) OR “reconstr*”[Text Word]) OR “coronally”[Text Word]) OR “laterally”[Text Word]) OR “matrix”[Text Word]) OR “transplant*”[Text Word]) AND “gingival recession”[MeSH Terms]) OR “gingival rec*”[Text Word]) OR “gingival exp*”[Text Word]) OR “periodontal plastic surgery” [Text Word]) OR “tissue graft” [Text Word]) OR “Collagen” [Text Word]) OR “Biomaterials” [Text Word]) OR “gingiva” [Text Word]) OR “keratin” [Text Word]) OR “transplantation” [Text Word]) OR “autologous” [Text Word]) OR “heterologous” [Text Word]) OR “xenograft” [Text Word]) OR “tissue regeneration” [Text Word]) OR “randomized controlled clinical trial” [Text Word]) OR “human” [Text Word].

#### 2.5.1 Selection of studies

Two independent reviewers (M.T. and A.A.) screened the titles and abstracts initially, then, full‐text articles were analyzed to decide whether the studies met the inclusion criteria. Disagreement between the reviewers was resolved through discussion until consensus was reached. Cohen's Kappa score was used to assess inter‐reviewer agreement of selection process (McHugh, 2012). The reasons for excluding studies were recorded.

#### 2.5.2 Data synthesis

Eligible studies underwent data extraction and validity assessment. Predesigned extraction forms were developed to retrieve and assess essential information such as title, authors, year, study location, study design, method of randomization, duration of study, allocation concealment, blinding (participants, investigators, and outcome assessors) length of observation period, and reported clinical outcomes.

Data synthesis was preformed through organizing data in an evidence table, and a descriptive summary was created to determine study characteristics, study quality, and results. Descriptive statistical analysis according to the mean values was used to evaluate the outcomes of test and control groups. Any disagreements were resolved by discussion.

#### 2.5.3 Risk of bias assessment

The assessments of the risk of bias for the included clinical trials were performed using the Cochrane Collaboration's tool for assessing risk of bias (Higgins et al., 2011). The analysis of each clinical trial was based on the following seven main domains: random sequence generation, allocation concealment, blinding participants and personnel, blinding of outcome assessment, incomplete outcome data, selective outcome reporting, and other sources of bias. The risk of bias was graded as low, high, or unclear for each domain based on the criteria defined in the Cochrane Handbook for Systematic Reviews of Interventions version 5.1.0 (Higgins and Green, 2011).

### 2.6 Data analysis

Data were analyzed using MedCalc for Windows version 15.0 (MedCalc Software, Ostend, Belgium). Descriptive statistics (mean and standard deviation) were used to describe the quantitative outcome variables (RD, RW, CRC, MRC, PD, CAL, and KTW). Meta‐analysis was carried out by combining the mean difference values for each of the seven outcome variables. The relative risk (RR) and standardized mean difference (SMD) as a pooled effect and a cut‐off values of 0.2 as small effect, 0.5 as medium effect, and 0.8 and more as larger effect (Cohen's rule) were used to report the overall effect, and student's *t* test was used to report the statistical significance. Cochran's Q, which is the weighted sum of squares on a standardized scale, was used to identify the presence of heterogeneity along with I^2^, which its percentage (0% to 100%) of observed total variation across included studies in meta‐analysis, due to real heterogeneity rather than chance. A value of I^2^, which is greater than 50%, was used to indicate increasing levels of unexplained variability in the effect sizes. Both the fixed and random effect models were used to obtain the pooled estimates of all the outcome variables. Based on the values of I^2^, appropriate overall effect (RR and SMD) was used to report its statistical significance. The results of different studies with its 95% confidence intervals (CI) and the overall effect (under the fixed and random effects model) with 95% CI were illustrated in Forest plots. A *p* value of <.05 and 95% CI were used to report statistical significance and its precision.

## 3 RESULTS

**Figure 1:**
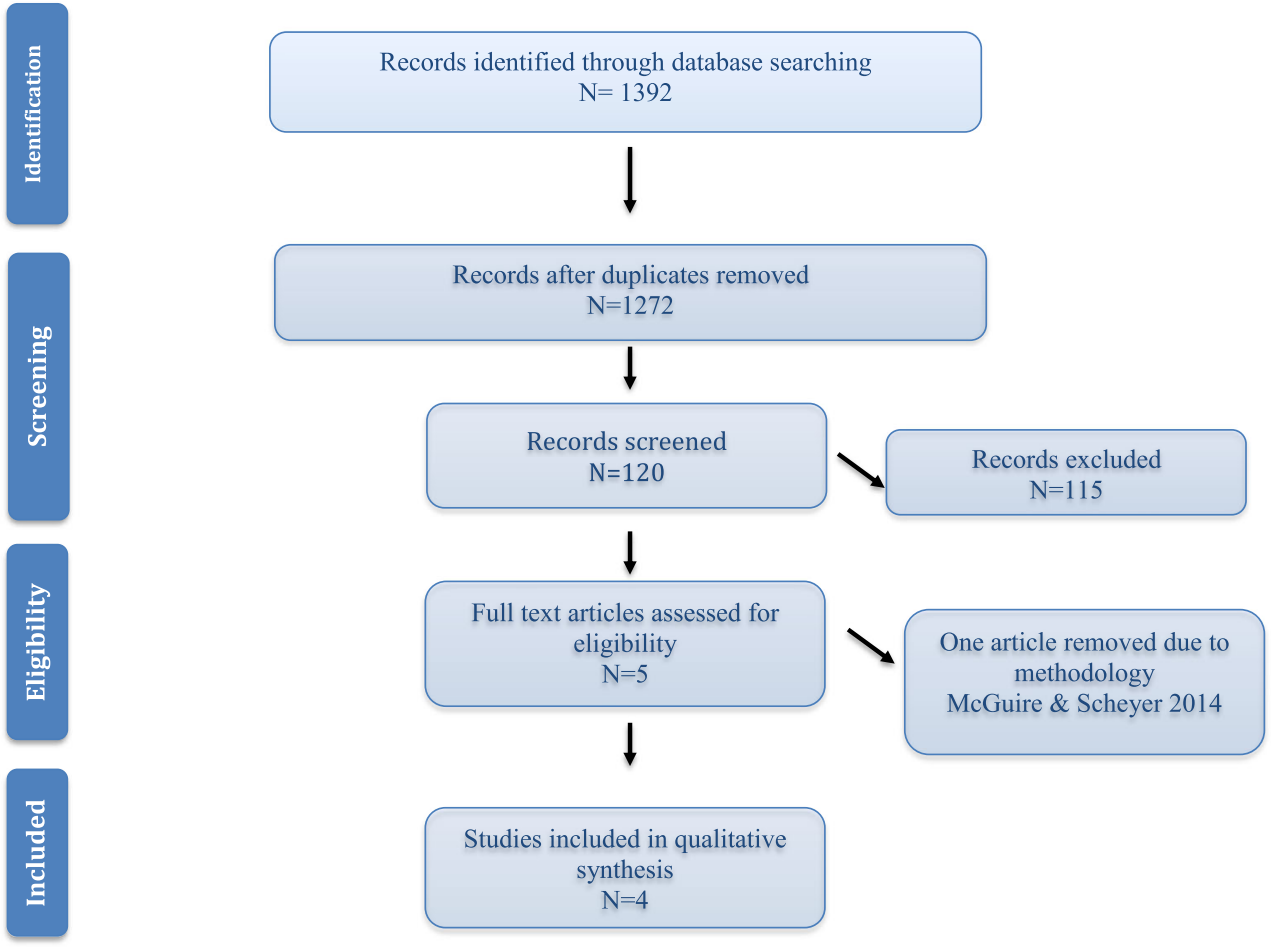
Study identification flow chart according to the Preferred Reporting Items for Systematic Reviews and Meta‐Analyses

The screening process according to Preferred Reporting Items for Systematic Review and Meta‐Analysis guidelines (Moher et al., 2009) is shown in Figure 1. A total of 1,392 studies were identified based on the search terminology from the various search engines. However, most of the studies (*N* = 1272) were duplicate results. The remaining 120 articles were screened, and 116 were excluded owing to lack of relevance and criteria applied. Assessments were performed for four included studies. The kappa value for inter‐reviewer agreement for potentially relevant articles was 0.95 for full text articles (Cohen, 1968).

### 3.1 Features of the included studies

#### 3.1.1 Study design and patient features

The age of the participants ranged from 18 to 50 years with a follow‐up period ranging from 3 months to 5 years. All included studies (Table 2) compared CMX with CTG for the treatment of MAGR (Aroca et al., 2013; Cieślik‐Wegemund, Wierucka‐Młynarczyk, Tanasiewicz, & Gilowski, 2016; Pietruska, Skurska, Podlewski, Milewski, & Pietruski, 2019; Tonetti et al., 2018).

**Table 2:** Characteristics of the clinical trials

Study	Study design	Follow‐up (months)	Inclusion criteria	Treatment in test group	Treatment in control group	Clinical outcomes (test vs. control) and authors conclusion
Pietruska et al. (2018) Poland	Randomized clinical trial (split mouth design)	12 months	*N* = 20 patients	MCAT + CMX	MCAT+CTG	Primary: Δ RD: 0.9 ± 0.7 mm vs. 1.5 ± 0.6 mm (*p* < 0.05)*
			Gender: 13 F:7 M			Δ RW: 0.8 ± 1.1 mm vs. 2.2 ± 1.2 mm (*P* < 0.05)*
			Age: 22–50 years (mean 47 years)			Δ MRC: 53.20 (32.17) % vs. 83.10 (27.63) % (*p* < .001)*
			Smokers: None			
			Teeth: Mandible			
			Recession: 91 Miller class I and II			Secondary: Δ PD: 0.10 ± 0.5 mm vs. 0.01 ± 0.5 mm (0.07)
						Δ CAL: 1.1 + 0.8 mm vs. 1.5 ± 0.6 mm (0.05)
						Δ KTW: 0.5 ± 0.7 mm vs. 2.8 ± 1.4 mm (*p* < .05)*
						• Statistically significant difference in RD, RW, KTW, and MRC favoring CTG group
Tonetti et al. (2018) Italy	Randomized clinical trial	6 months	*N* **=** 187 patients	CAF + CMX	CAF + CTG	Primary: Δ RD: 1.7 ± 1.1 mm vs. 2.1 ± 1.0 mm (95% Cl [0.25, –0.63])*
			Gender: 69 M: 118 F			Δ CRC: 48% vs. 70% (95% Cl [1.8, 8.8])*
			Age: 39–41.3 years (mean 40 years)			Secondary: Δ PD: 0.1 ± 0.7 mm vs. 0.3 ± 0.1 mm (95% Cl [0.31, –0.04])*
			Smokers: 51			Δ KTW: 0.1 ± 1.1 mm vs. 0.5 ± 1.2 mm (95% Cl [0.24, –0.70])*
			Teeth: 485			
			Recession: 485 Miller Class I and II Average baseline recession was 2.5 ± 1.0 mm			
						• Statistically significant difference in RD, CRC, PD, and KTW favoring CTG group

Wegemund et al. (2016) Poland	Randomized clinical trial	3 and 6 months	*N* = 28 patients	Tunnel + CMX	Tunnel + CTG	Primary: Δ RD: 2.6 ± 0.5 mm vs. 2.5 ± 0.8 mm (*p* < .001)**
			Gender: 9 M: 19 F			
			Age: 20–50 years (mean 35 years)			Δ RW: 2.9 ± 0.8 mm vs. 2.6 ± 0.8 mm (*p* > .05)
			Teeth: Mandible and Maxilla			Δ CRC: 83%. vs. 70% (*p* > .05)
			30 incisors, 15 premolars, and 1			Δ ARC: 91% ± 13 vs. 95% ± 11 (0.027)
			molar in the control group and			Secondary: Δ CAL: 2.6 ± 0.3 mm vs 2.6 ± 0.4 mm (*p* > .05)
			33 incisors and canines, 18 premolars,			Δ KTW: 1.7 ± 1.5 mm vs. 1.4 ± 1.2 mm (*p* > .05)
			and 5 molars in the test group.			• Statistically significant difference in RD favoring CMX group
			Recession: 106 Miller Class I and II			
Aroca et al. (2013) Hungary	Randomized clinical trial (split mouth design)	12 months	*N* = 22 patients	MCAT + CMX	MCAT + CTG	Primary: Δ RD: 1.3 ± 0.5 mm vs. 1.6 ± 0.4 mm (*p* < .05)*
			Gender: Not mentioned			Δ RW: 2.4 ± 1.0 mm vs. 3.3 ± 0.9 mm (*p* < .05)*
			Age: 18 years and more			Δ CRC: 42% vs. 85% mm (*p* < .05)*
			Teeth: Maxilla and Mandible (Anterior, Premolar, and Molar)			Δ MRC: 71 ± 21% vs. 90 ± 18% (*p* < .05)*
						Secondary: Δ PD: 0.0 ± 0.3 mm vs. 0.0 ± 0.2 mm (0.374)
			Recession: 165 Miller class I and II			Δ CAL: 1.3 + 0.6 mm vs. 1.7 ± 0.4 mm (*p* < .05)*
						Δ KTW: 0.3 ± 0.7 mm vs. 0.7 ± 0.7 mm (0.079)
						Statistically significant difference in RD, RW, RC, and CAL favoring CTG group

Abbreviations: CAL, clinical attachment level gain; CMX, xenogeneic collagen matrix; CRC, complete root coverage; CTG, connective tissue graft; MCAT, modified coronally advanced tunnel; MRC, mean root coverage; PD, probing depth; Δ RD, recession depth; RW, recession width; KTW, keratinized tissue width.

Statistically Significant difference favoring CTG group*

Statistically Significant difference favoring CMX group**

#### 3.1.2 Sites, recession, and defect characteristics

All studies clearly included MAGR with Class I and II Miller's GR. Three studies involved both maxillary and mandibular teeth (Aroca et al., 2013; Cieślik‐Wegemund et al., 2016; Tonetti et al., 2018), whereas one study reported only mandibular teeth (Pietruska et al., 2019). The remaining did not mention which arches were treated. Regarding the type of teeth treated, two studies included anterior teeth, premolars, and molars (Cieślik‐Wegemund et al., 2016; Pietruska et al., 2019); whereas, the remaining studies did not mention the type of teeth treated in a detailed manner.

### 3.2 Type of interventions

GRs were surgically treated by CTG + CAF in control groups of all included clinical trials. Autologous CTGs were harvested from the palate using the trap door technique whenever feasible and the de‐epithelialized free gingival graft in cases with insufficient tissue thickness as described in most articles (Aroca et al., 2013; Cieślik‐Wegemund et al., 2016; Tonetti et al., 2018). The CTG was immediately harvested after tunnel preparation by using either a modified distal wedge procedure (Azzi, Etienne, & Carranza, 1998) or the single‐incision technique (Hürzeler & Weng, 1999) depending on anatomical considerations. If needed, the harvested graft was trimmed using a N°15 blade to achieve an optimal thickness of 1–1.5 mm.

Recessions were also surgically treated with a variation of techniques. One study performed CAF (Tonetti et al., 2018) whereas other two performed modified coronally advanced tunnel (MCAT; Aroca et al., 2013; Pietruska et al., 2019). Only one study reported the application of TUN to cover both grafts (Cieślik‐Wegemund et al., 2016). The flaps were positioned coronally to the cemento‐enamel junction by means of suspended sutures placed above the contact point (Azzi et al., 1998).

### 3.3 Postoperative care

In terms of postoperative management, all patients in three studies reported prescription of chlorhexidine mouth rinse after the surgery with a usage range between 5 and 21 days with two different concentrations either 0.2% (Aroca et al., 2013; Pietruska et al., 2019) or 12% (Cieślik‐Wegemund et al., 2016). On the other hand, one study instructed their patients to use 0.5% chlorhexidine gel on the first week (Tonetti et al., 2018). In terms of antibiotic use, Tonetti et al. (2018) reported the use of antibiotics; however, specific details were not mentioned. Another study used Augmentin 625 mg for 7 days due to their university regulations (Aroca et al., 2013). With regard to analgesics use, one study reported that patients were given analgesics 50‐mg Cataflam for 3 days (Aroca et al., 2013); whereas in another study, patients were given 600‐mg ibuprofen or 500‐mg paracetamol (Tonetti et al., 2018). Finally, one trial reported instructing their patients to use analgesics when needed, but details were not mentioned (Pietruska et al., 2019).

### 3.4 Risk of bias assessment

**Figure 2:**
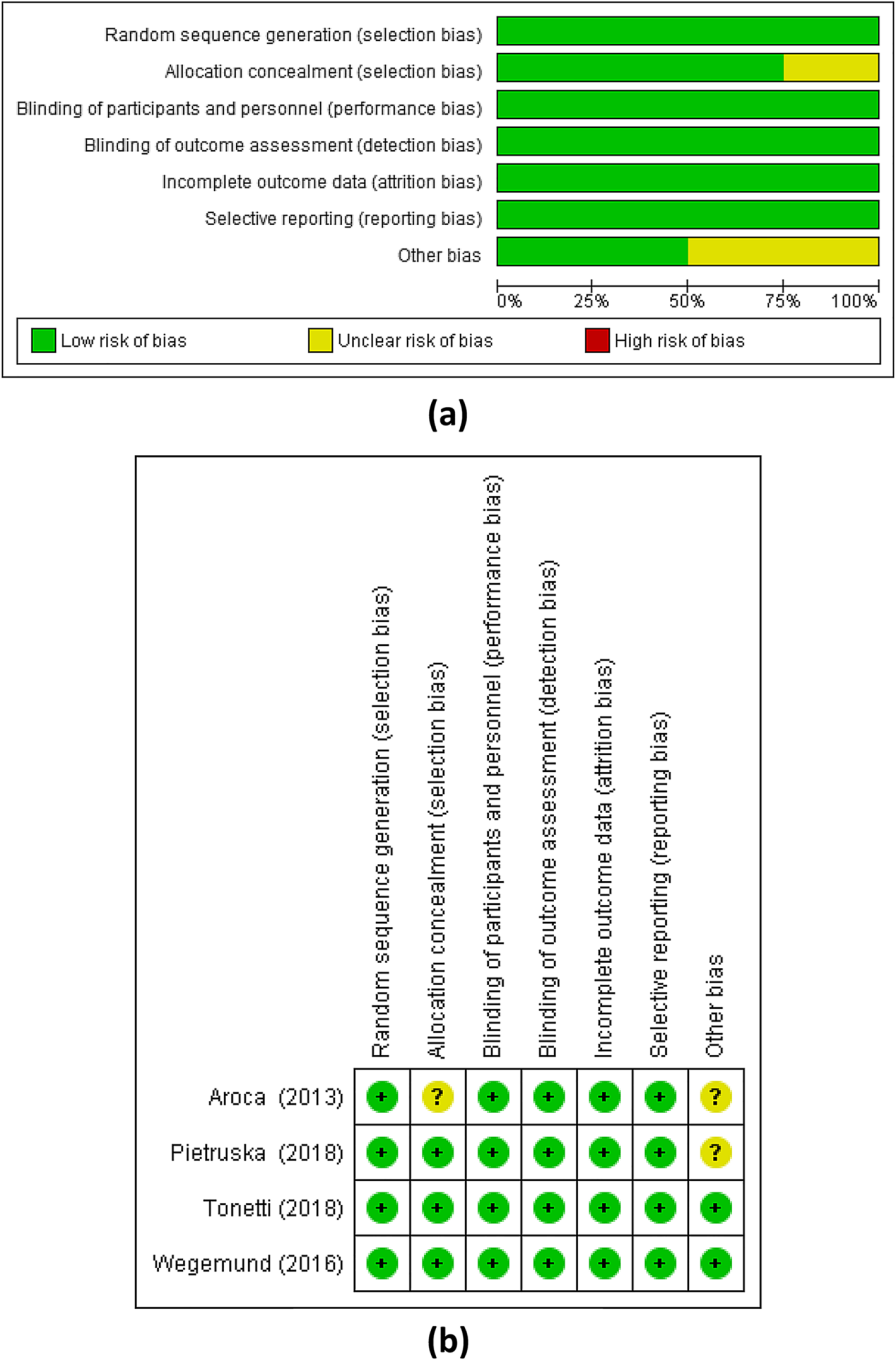
Risk of bias assessments (a) low, unclear, and high risks (b) summary of risks in the included studies

The results of bias assessment of the included studies are presented in Figure 2.,b. None of the studies obtained the highest score in the quality analysis. Allocation concealment was clearly mentioned in three studies (Aroca et al., 2013; Cieślik‐Wegemund et al., 2016; Tonetti et al., 2018). Blinding was not performed in two of the included studies (Aroca et al., 2013; Cieślik‐Wegemund et al., 2016). Furthermore, incomplete outcome data and selective outcome reporting were found in two of the studies (Cieślik‐Wegemund et al., 2016; Tonetti et al., 2018). Only one study assessed the similarity between groups at baseline and statistically controlled the confounding and interaction factors (Tonetti et al., 2018). None of the studies reported adherence to the CONSORT statement recommendations as this can bring these studies to uncertain risk of bias (Moher et al., 2001).

### 3.5 Outcomes measured

#### 3.5.1 Primary outcomes

- Recession depth (RD)

**Figure 3:**
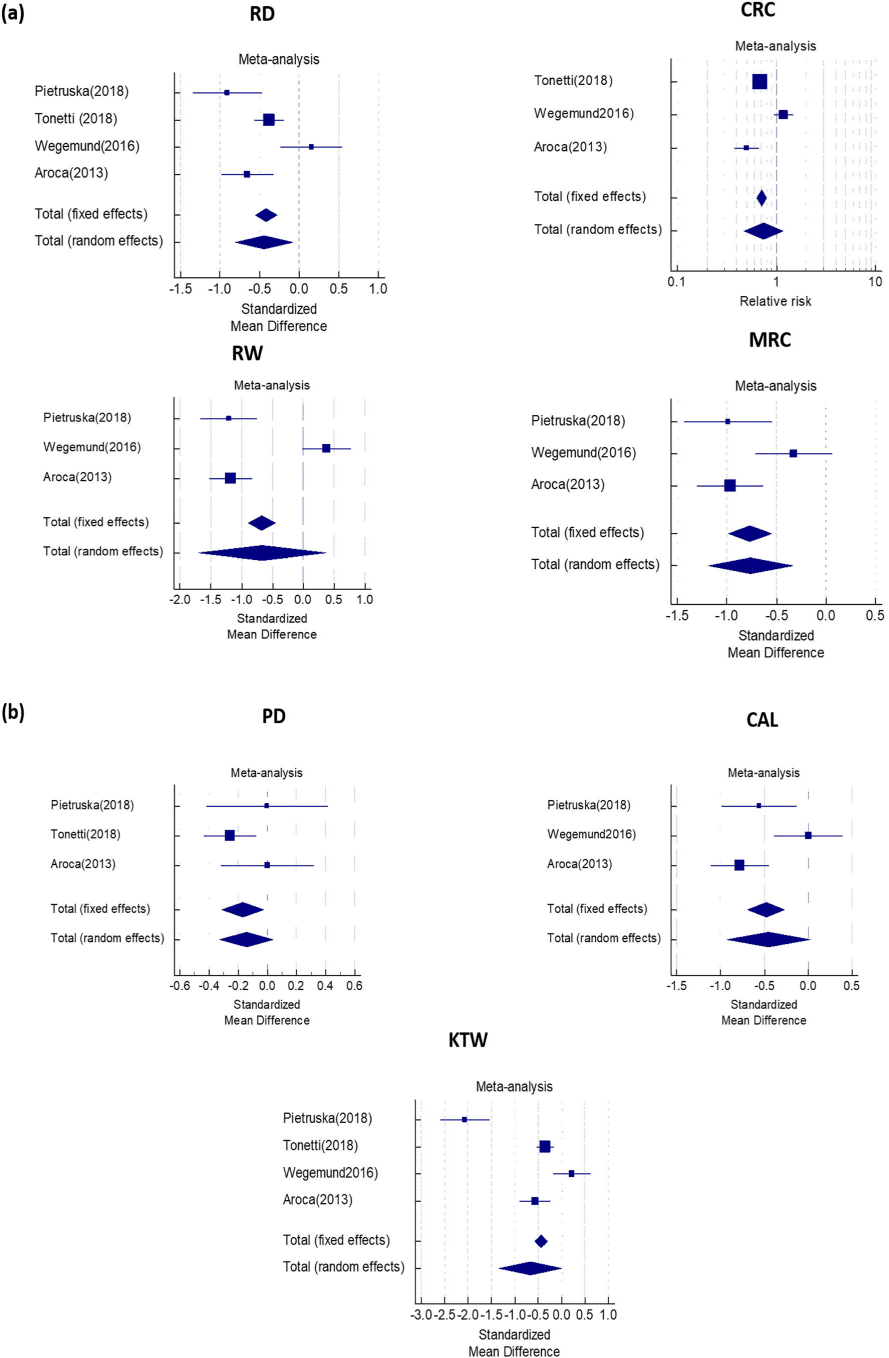
Forest plots for the primary and secondary outcome variables derived from meta‐analyses (a) recession depth, recession width, complete root coverage, mean root coverage; (b) probing depth, clinical attachment level, keratinized tissue width

The Cochran's Q, which is the weighted sum of squares on a standardized scale, is statistically significant (Q = 16.07, *p* < .0011) with a high I^2^ value (81.33%), which implies heterogeneity across the four studies. CMX had significantly lower mean values of RD when compared with control group (CTG group) (SMD = −0.442, *t* = −2.402, *p* = .017). The overall effect is medium effect (Table 3, Figure 3.).

- Recession width (RW)

Across the three eligible studies, a random effect was considered where both Cochran's Q (42.55) and I^2^ value (95.3%) were high. The pooled estimate shows no statistically significant difference in the mean values of RW between the CMX and CTG (SMD = −0.669, *t* = −1.275; *p* = .203; Table 3, Figure 3.).

- Mean root coverage (MRC)

The Cochran's Q value is statistically significant (Q = 7.488, *p* = .0237), and I^2^ value (73.29%) was high, which implies high heterogeneity across the three studies. As a result, the pooled SMD by random effect could be used to infer that CMX is having significantly lower mean values of MRC when compared with CTG (SMD = −0.760, *t* = −3.510, *p* = .001; Table 3, Figure 3.). The overall effect is larger effect.

- Complete root coverage (CRC)

The Cochran's Q (Q = 27.40, *p* < .0001) and I^2^ value (92.70%) were high, which implies high heterogeneity across the three studies. As a result, the pooled effect size (RR) by random effect was used to infer that, CMX has not achieved statistically significant CRC when compared with CTG (Pooled RR = 0.743, z = −1.281, *p* = .200; Table 4, Figure 3.).

**Table 3:** Meta‐analysis for outcome variables: RD, RW, and MRC

RD	CMX group			CTG group			SMD	95% CI	
Study		Total	Mean (SD)		Total	Mean (SD)			
Pietruska (2018)	MCAT + CMX	46	0.9 (0.7)	MCAT + CTG	45	0.15 (0.6)	−0.912	−1.346, −0.477	
Tonetti (2018)	CAF + CMX	242	1.7 (1.1)	CAF + CTG	243	2.1 (1)	−0.380	−0.560, −0.200	
Wegemund (2016)	Tunnel + CMX	59	2.6 (0.5)	Tunnel + CTG	47	2.5 (0.8)	0.153	−0.233, 0.538	
Aroca (2013)	MCAT + CMX	78	1.3 (0.5)	MCAT + CTG	78	1.6 (0.4)	−0.659	−0.983, −0.336	
**Overall effect**								Weight (%)	
Fixed effects: Total *N* = 838; SMD = −0.417 (95% CI [−0.554, −0.279]); *t* value = −5.952; *p* < .001								Fixed	Random
Random effects: Total *N*: 838; SMD = −0.442 (95% CI [−0.804, −0.0809]); *t* value = −2.402; *p* = .017								10.25	21.90
Test for heterogeneity: Q = 16.07; *p* = .0011; I^2^ = 81.33% (95% CI [51.33, 92.84])								58.51	29.36
								12.96	23.41
								18.28	25.33

RW	CMX group			CTG group			SMD	95% CI	
Study		Total	Mean (SD)		Total	Mean (SD)			
Pietruska (2018)	MCAT+CMX	46	0.8 (1.1)	MCAT + CTG	45	2.2(1.2)	−1.207	−1.656, −0.757	
Wegemund (2016)	Tunnel+CMX	59	2.9 (0.8)	Tunnel + CTG	47	2.6(0.8)	0.372	−0.0159, 0.761	
Aroca (2013)	MCAT+CMX	78	2.4 (1.0)	MCAT + CTG	78	3.3(0.4)	−1.176	−1.517, −0.835	
**Overall effect**								Weight (%)	
Fixed effects: Total *N* = 183; SMD = −0.673(95% CI [−0.894, −0.452]); *t* value = −5.988; *p* < .001								Fixed	Random
Random effects: Total *N*: 183; SMD = −0.669 (95% CI [−1.702, 0.363); *t* value = −1.275; *p* = .203								24.67	32.88
Test for heterogeneity: Q = 42.55; *p* < .0001; I^2^ = 95.30% (95% CI [89.54, 97.89])								32.97	33.39
								42.37	33.74

MRC	CMX group			CTG group			SMD	95% CI
Study		Total	Mean (SD)		Total	Mean (SD)		
Pietruska (2018)	MCAT + CMX	46	53.2 (32.2)	MCAT + CTG	45	83.1 (27.6)	−0.988	−1.426, −0.550
Wegemund (2016)	Tunnel + CMX	59	91 (13)	Tunnel + CTG	47	95 (11)	−0.327	−0.714, 0.0608
Aroca (2013)	MCAT + CMX	78	71 (21)	MCAT + CTG	78	90 (18)	−0.967	−1.300, −0.634
**Overall effect**							Weight (%)	
Fixed effects: Total *N* = 353; SMD = −0.768 (95% CI [−0.985, −0.550]); *t* 92.04) value = −6.951; *p* < .001							Fixed	Random
Random effects: Total N: 353; SMD = −0.760 (95% CI [−1.186, −0.334]); *t* value = −3.510; *p* = .001							25.10	30.98
Test for heterogeneity: Q = 7.488; *p* = .0237; I^2^ = 73.29% (95% CI [10.40, 92.04])							31.94	33.27
							42.96	35.75

Abbreviations: CMX, xenogeneic collagen matrix; CTG, connective tissue graft; MCAT, modified coronally advanced tunnel; MRC, mean root coverage; RD, recession depth; RW, recession width.

**Table 4:** Meta‐analysis for outcome variable complete root coverage (CRC)

Study	CMX group		CTG group		Relative risk	95% CI	z	P	Weight (%)	
	No. CRC/N		No. CRC/N						Fixed	Random
Tonetti (2018)	CAF + CMX	116/242	CAF + CTG	170/243	0.685	0.587 to 0.800	—	—	55.04	34.72
Wegemund (2016)	Tunnel+CMX	49/59	Tunnel + CTG	33/47	1.183	0.950 to 1.472	—	—	27.58	33.37
Aroca (2013)	MCAT+CMX	33/78	MCAT + CTG	66/78	0.500	0.379 to 0.659	—	—	17.38	31.91
Total (fixed effects)	—	198/379	—	269/368	0.707	0.629 to 0.795	−5.807	<.001	100.00	100.00
Total (random effects)	—	198/379	—	269/368	0.743	0.472 to 1.170	−1.281	.200	100.00	100.00
Test for heterogeneity: Q = 27.40; df = 2; *p* < .0001; I^2^ (inconsistency) = 92.70%; 95% CI for I^2^ = 81.97% to 97.05%										

Abbreviations: CMX, xenogeneic collagen matrix; CRC, complete root coverage; CTG, connective tissue graft.

A summary of meta‐analysis for primary outcome variables along with the corresponding Forest plots is presented in Tables 3, 4, and Figure 3., respectively.

### 3.6 Secondary outcomes

- Probing depth (PD)

Change in “PD” was reported in three studies (Aroca et al., 2013; Cieślik‐Wegemund et al., 2016; Pietruska et al., 2019). The Cochran's Q is lower and not statistically significant (Q = 2.682, *p* = .262) and I^2^ value (25.43%) also lower, which implies lack of heterogeneity across the three studies. Hence, the pooled SMD by fixed effect was used to infer that CMX is having significantly lower mean values of PD when compared with CTG (SMD = −0.169, *t* = −2.285, *p* = 0.023). The overall effect is small effect (Table 5, Figure 3b).

- Clinical attachment level (CAL)

“CAL” was documented in three studies as well (Aroca et al., 2013; Cieślik‐Wegemund et al., 2016; Pietruska et al., 2019). Random effect was considered where both Cochran's Q (9.56) and I^2^ value (79.09%) were high. The pooled estimate shows no statistically significant difference in the mean values of CAL between CMX and CTG (SMD = −0.452, *t* = −1.888, *p* = .060; Table 5, Figure 3b).

- Keratinized tissue width (KTW)

Across the four eligible studies, Cochran's Q value was high and statistically significant (Q = 52.742, *p* < .0001) also the I^2^ value (94.31%) was high, which implies high heterogeneity. As a result, the pooled SMD by random effect was used to infer that no statistically significant difference in the mean values of KTW between CMX and CTG (SMD = −0.665, *t* = −1.949, *p* = .052).

**Table 5:** Meta‐analysis for outcome variables: PD, CAL, and KTW

PD	CMX group			CTG group			SMD	95% CI	
Study		Total	Mean (SD)		Total	Mean (SD)			
Pietruska (2018)	MCAT + CMX	46	0.10 (0.5)	MCAT + CTG	45	0.10 (0.50	−0.00198	−0.415, 0.411	
Tonetti (2018)	CAF + CMX	242	0.10 (1.1)	CAF + CTG	243	0.30 (0.1)	−0.256	−0.435, −0.077	
Aroca (2013)	MCAT + CMX	78	0.0 (0.3)	MCAT + CTG	78	0.0 (0.2)	0.000	−0.315, 0.315	
**Overall effect**								Weight (%)	
Fixed effects: Total *N* = 732; SMD = −0.169 (95% CI [−0.314, −0.024]); *t* value = −2.285; *p* = .023								Fixed	Random
Random effects: Total *N* = 732; SMD = −0.143 (95% CI [−0.327, −0.0403]); *t* value = −1.532; *p* = .126								12.63	17.32
Test for heterogeneity: Q = 2.682; *p* = .262; I^2^ = 25.43% (95% CI [0.00, 97.50])								65.87	55.93
								21.50	26.75

CAL	CMX group			CTG group			SMD	95% CI	
Study		Total	Mean (SD)		Total	Mean (SD)			
Pietruska (2018)	MCAT + CMX	46	1.1 (0.8)	MCAT + CTG	45	1.5 (0.6)	−0.560	−0.981, −0.139	
Wegemund (2016)	Tunnel + CMX	59	2.6 (0.3)	Tunnel + CTG	47	2.6 (0.4)	0.000	−0.385, 0.385	
Aroca (2013)	MCAT + CMX	78	1.3 (0.6)	MCAT + CTG	78	1.7 (0.4)	−0.781	−1.107, −0.454	
**Overall effect**								Weight (%)	
Fixed effects: Total *N* = 353; SMD = −0.480 (95% CI [−0.693, −0.268]); *t* value = −4.439; *p* < .001								Fixed	Random
Random effects: Total *N* = 353; SMD = −0.452(95% CI [−0.924, 0.019]); *t* value = −1.888; *p* = .060								26.06	31.75
Test for heterogeneity: Q = 9.56; *p* = .008; I^2^ = 79.09% (95% CI [33.21, 93.45])								31.09	33.08
								42.84	35.17

KTW	CMX group			CTG group			SMD	95% CI	
Study		Total	Mean (SD)		Total	Mean (SD)			
Pietruska (2018)	MCAT + CMX	46	0.5 (0.7)	MCAT + CTG	45	2.8 (1.4)	−2.067	−2.581, −1.554	
Tonetti (2018)	CAF + CMX	242	0.1 (1.1)	CAF + CMX	243	0.5 (1.2)	−0.347	−0.526, −0.167	
Wegemund (2016)	Tunnel + CMX	59	1.7 (1.5)	Tunnel + CTG	47	1.4 (1.2)	0.217	−0.169, 0.603	
Aroca (2013)	MCAT + CMX	78	0.3 (0.7)	MCAT + CTG	78	0.7 (0.7)	−0.569	−0.890, −0.247	
**Overall effect**								Weight (%)	
Fixed effects: Total *N* = 838; SMD = −0.444 (95% CI [−0.583, −0.305]); *t* value = −6.263; *p* < .001								Fixed	Random
Random effects: Total *N* = 838; SMD = −0.665 (95% CI [−1.335, 0.004]); *t* value = −1.949; *p* = .052								7.53	23.36
Test for heterogeneity: Q = 52.742; *p* < .0001; I^2^ = 94.31% (95% CI [88.55, 97.17])								60.20	26.45
								13.26	24.79
								19.01	25.41

Abbreviations: CAL, clinical attachment level; CMX, xenogeneic collagen matrix; CTG, connective tissue graft; KTW, keratinized tissue width; MCAT, modified coronally advanced tunnel; PD, probing depth.

A summary of meta‐analysis for secondary outcome variables along with the corresponding Forest plots is presented in Table 5 and Figure 3.b, respectively.

### 3.7 Aesthetics, healing, and pain

Pietruska et al. (2019) measured the esthetic outcome using Root Coverage Esthetic Score (RES) proposed by Cairo, Rotundo, Miller, and Pini Prato (2009). The average RES in CMX group was 7.11 ± 1.95 and 8.36 ± 1.78 in CTG group. There was a statistically significant difference in the RES favoring CTG group (*p* < .001). However, gingival color was comparable in both groups. Cieślik‐Wegemund et al. (2016) reported significantly greater pain and swelling in the first week after surgery in CMX group, which subsided afterwards. In addition, authors reported a statistically significant better color match in CMX group at 6‐month follow‐up. Furthermore, upon patient satisfaction evaluation 12 months after surgery, Aroca et al. demonstrated that the number of subjects who reported 100% satisfaction was higher in the CMX group compared with CTG; however, the difference was not statistically significant (*p* > .05; Aroca et al., 2013). Tonetti et al. measured the time to recovery of surgery area using OHIP‐14 questionnaire (Slade, 1997). They reported that time of recovery was 1.8 days shorter in CMX group compared with CTG group. This difference was shown to be statistically significant. Finally, they also reported that CMX group surgery was 15.7 min shorter (95% CI from 11.9 to 19.6, *p* < .0001) and less painful as reported by patient (11.9 VAS units, 95% CI from 4.6 to 19.1, *p* = .0014) in CMX subjects (Tonetti et al., 2018). A summary is presented in (Table 2).

## 4 DISCUSSION

Novel options for the treatment of GR are one of the priority areas in periodontal practice especially in the presence of numerous defects. An effort to reduce the number of surgeries and intraoral surgical sites, together with the need to satisfy the patient's esthetic desire, is always an area of intensive research in dentistry. To date, however, the research related to the efficacies of various treatment modalities in MAGR remains debatable. So far, there is limited evidence to show the efficacy of any one type of procedure (Graziani et al., 2014). Collagen matrix of porcine and bovine origin has been developed to be a safe alternative material, which provides regeneration of gingival tissues and promotes wound healing (Sanz et al., 2009; Thoma, Sancho‐Puchades, Ettlin, Hämmerle, & Jung, 2012). Animal studies have shown that CMX is replaced with the host's own tissue with the desired histologic and functional characteristics (Thoma et al., 2012). Nowadays, CMX is increasingly being used for its effectiveness in achieving root coverage, reduction of recession, and gain in tissue thickness. In the current review, studies comparing CMX with CTG in multiple adjacent defects were analyzed with the objective to understand the improvement in clinical parameters related to the treatment of periodontal plastic procedures used in oral surgical interventions.

The efficacy of CMX in covering recession defects compared with the current gold standard, CTG showed 84% and 89% root coverage at 6 months and 1 year, respectively with CMX + CAF. However, better results were achieved with CAF + CTG with 97% and 99% root coverage at 6 months and 1 year, respectively (McGuire & Scheyer, 2010). Also, the noninferiority of CMX + CAF compared with CTG + CAF in achieving root coverage in MAGR, alongside shorter surgical procedure and recovery period, has been presented (Tonetti et al., 2018). The use of CMX + CAF as an alternative to CAF + CTG (Cardaropoli, Tamagnone, Roffredo, & Gaveglio, 2012) for GR indicated that the former resulted in MRC of 94% versus 97%. A trial with CMX + CAF versus CAF alone showed similar improvements in MRC favoring CMX with significant variations in gingival thickness and KT gain between both groups (Jepsen et al., 2013). On similar lines, Cardaropoli et al. indicated that CMX + CAF demonstrated superior results compared with CAF alone in root coverage (93.25% vs. 81.49%) and CRC (72% vs. 58%; Cardaropoli, Tamagnone, Roffredo, & Gaveglio, 2014). Furthermore, Cairo et al. (2017) reported a comparable result in terms of soft tissue augmentation between CMX and CTG around dental implants with better aesthetics and patient comfort. This review indicated that the clinical outcomes of CMX were noninferior to CTG.

The use of the extended flap technique promoted better vascularization in the center of the flap, due to flap extension and avoided its contraction in the healing period. Owing to this, the test group showed greater root coverage than the control group. This was indicated in the study conducted by Reino et al. (2015), where root coverage after 3 months was superior for the test group (82.33%) compared with the control group (60.78%); this phenomenon was maintained after 6 months. Moreover, a greater reduction in height and width of GRs was found compared with the control group at 3 and 6 months. Another study indicated that for treating MAGR, tunnel technique could be used to achieve early healing and good aesthetics and improved blood supply (Aroca et al., 2013). However, poor visibility of the inner recipient tissue limits the widespread use of this procedure. A modified coronally advanced tunnel (MCAT) using either CMX or CTG indicated that compared with baseline, both treatments resulted in statistically significant root coverage, but CMX yielded lower (CRC) compared with CTG. CRC was found at 42% of test sites and at 85% of control sites, respectively. The relatively low percentage of CRC may be attributed to involving posterior teeth for both test and control sites, which pose a significant anatomical challenge.

Moreover, in terms of postoperative healing, Cieślik‐Wegemund et al. (2016) indicated that healing with no complications, such as allergic reactions, infections, or matrix exfoliation were observed using CMX. In fact, color match was found better than CTG after 1 year of the surgery. The authors concluded that when CMX was compared with CTG in root coverage, both methods were effective in reducing clinical parameters such as RD and width after surgery compared with the baseline measurements. The mean width of the KT increased in both groups. The mean average root coverage after 6 months was 95% in the control group and 91% in the test group (*p* < .05), and the percentage of patients with complete coverage of all recessions was 71.4% in the control group and 14.3% in the test group (*p* < .05).

It seems that CTG provide better KTW, although this was not significant (*p* = .052) probably due to the limited number of studies included. A possible explanation for the difference in KTW is the lack of cells of the CMX (Yu, Tseng, & Wang, 2018). However, it is important to note that CMX was found to be completely incorporated into the adjacent host connective tissues in the absence of a significant inflammatory response. The healing was characterized by the formation of new cementum and new connective tissue attachment in the apical aspect of the defect and by a junctional epithelium in its most coronal third. When compared with CAF alone, both techniques rendered similar clinical outcomes. Although the CMX graft attained more tissue regeneration, with a shorter epithelium and a larger new‐cementum formation (Vignoletti et al., 2011).

The authors are aware of the limitations of the current review. Only four randomized controlled trials were found with matched criteria. We believe that clinical experience and availability of materials contributed to this limitation. In addition, heterogenicity was obvious among included studies with different surgical techniques and variation of reported outcomes preventing a firm conclusion.

The studies identified in the current meta‐analysis were not conclusive about the superiority of one procedure over the other. However, in spite of clinical outcomes compared with CTG in the treatment of MAGR, CMX may be considered as noninferior alternative avoiding the need for second area of surgery and shortening the procedure time with reduced patient's discomfort postoperatively. Determination of surgical techniques may have a considerable role where TUN technique seems to enhance CRC (Cieślik‐Wegemund et al., 2016). Finally, long‐term studies are needed to identify elements that make CMX a successful alternative in MAGR.

## CONFLICT OF INTEREST

The authors report no conflicting interest with respect to this review article.

## CLINICAL RELEVANCE

Several studies have reported favorable outcomes of CMX in single GR. However, better understanding of the efficacy of CMX for the treatment of multiple adjacent gingival recessions (MAGRs) has been addressed in this review. Within the limited identified studies: heterogeneity in methodology, outcome measured, and data reported was evident. The average percentage of MRC for CMX and CTG was 65.8% and 84.5% respectively, indicating that CMX was not as effective as CTG in MAGRs. Furthermore, CMX showed favorable patient‐reported outcomes. Although CMX provided acceptable clinical outcomes, using it as an alternative to CTG for root coverage remains to be determined.
